# A Case Study Investigating the Relational Well-Being of International Students at Hohai University Nanjing, Jiangsu Province of China

**DOI:** 10.3390/bs14070544

**Published:** 2024-06-27

**Authors:** Haihua Ying, Abdul Rasool Khoso, Shahnaz Bhutto

**Affiliations:** 1 Students’ Service Center, International School, Hohai University, Xikang Road, No. 1, Gulou District, Nanjing 210024, China; 20120620@hhu.edu.cn; 2 Department of Sociology, School of Public Administration, Hohai University, Nanjing 210098, China; shahnaz.socio.95@gmail.com

**Keywords:** relational well-being, international students, challenges, Hohai University, Nanjing, China

## Abstract

This study acknowledges the growing importance of international student mobility and examines the relational well-being of international students at Hohai University in Nanjing, China. Understanding the complexities of interactions among international students is essential for their well-being and the university’s overall success, since this tendency continues to increase. By examining the distinct possibilities and problems faced by international students at Hohai University and considering elements including cultural distinctiveness, the campus environment, interpersonal dynamics, and support networks, the study fills a research void. For this purpose, 150 international students at Hohai University participated in semi-structured interviews and 10 participants participated in unstructured interviews as part of the mixed-methods approach to obtain in-depth information. Hence, the descriptive data were analyzed using SPSS and qualitative analyses were performed using NVIVO. According to preliminary findings derived from quantitative data, important results emphasize the significant impact of homesickness (0.143, T value: 5.931) and the positive correlation of relational well-being (0.146), highlighting their significance. The effect of the language barrier is also significant (0.125, T value: 4.378), whereas loneliness has little bearing (0.011, T value: 0.847). Additionally, the highest concern of the international students (M = 8.35; SD = 1.43) was making connections with local students. Additionally, (M = 8.21; SD = 1.15) international students favored Hohai University, which provided a welcoming atmosphere for intercultural dialogue. The Foreign Student Office, social events, cultural exchange programs, counseling services, and peer support networks are just a few examples of the support structures and networks crucial to international students’ general well-being. At the same time, the cultural hurdles, including the language barrier and loneliness, seemed to be the challenge. The study concludes by shedding light on the relational well-being of international students at Hohai University and highlighting the importance of community-building and supporting actions. The results provide insightful information that will help the institution better respond to the particular difficulties that international students encounter, creating a more welcoming and stimulating atmosphere. This study establishes the foundation for further research in comparable contexts and advances our understanding of relational well-being in the context of experiences for overseas students.

## 1. Introduction

The trend of students traveling to other countries for educational purposes has significantly increased, becoming an essential element in global migration with an annual growth rate between 7% and 10% [1,2]. According to the Organization for Economic Co-operation and Development (OECD) (2013), those who go beyond national borders to further their education are called international students [3]. International students contribute to a variety of host communities and are frequently seen as a symbol of academic excellence [4]. In addition to enhancing national economies, overseas students are a significant source of skilled labor after graduation [5,6]. Ledger’s research in Australia demonstrates the significant economic impact that overseas students make [7]. Similarly, Xu and Stahl highlight the necessity for Chinese higher education institutions to provide high-quality reciprocal services while highlighting the financial effect of international students on the country’s economy [8]. In exchange for their financial commitment, students are guaranteed to get the best possible social and educational experiences in this reciprocal partnership [9]. International students encounter several difficulties that might compromise their general well-being in host countries despite their cultural and economic contributions [10]. Studies from the past and present continually note loneliness as a significant problem [11,12]. Wawera and McCamley found that loneliness is frequently made worse by obstacles to establishing new relationships within host communities [12]. To improve the well-being of international students, a more thorough knowledge of their relationship experiences is required, considering the significant contributions they make to host cultures. The importance of this study stems from the understanding that favorable interpersonal interactions benefit international students’ well-being and the university’s general performance and standing. Existing research has pointed out several issues, such as the possibility that loneliness among international students would worsen [12]. However, little is known about Hohai University’s particular setting or the particular interpersonal difficulties and developments that its community of international students face. Therefore, this research was conducted to evaluate and give insightful information to other researchers to help them know more about their institutional education-based system and relational well-being. Thus, this study seeks to explore the current state of relational well-being and challenges and opportunities for improving the relational aspects of the international student experience among Hohai students. 

### 1.1. Relational Well Being

Positive relationships with others, as shown by Cogan et al., are highlighted as a critical aspect contributing to individuals’ eudemonic well-being in seminal works [13]. The academic and post-school vocational achievements of immigrant students are boosted by positive school interactions [14]. However, because they are frequently cut off from established social groups in their home countries, immigrant students’ well-being may be compromised as they struggle to develop ties with native students [15]. It can be challenging to navigate new places without the support of close friends and family. It makes sense to conceptualize relational well-being from a eudemonic standpoint since this enables a robust conception of well-being that can withstand the difficulties of being an international student. Eudemonic well-being, which revolves around reaching one’s potential and leading a purposeful life, depends on relational well-being, which fosters meaningful and positive relationships. Eudemonic well-being depends on personal development and life satisfaction, both facilitated by supportive relationships [16]. It looks at how connections might lessen the challenges a student faces along the way. Positive well-being outcomes, promoted by interactions and connections with other students, are another definition of relational well-being in this study.

### 1.2. The Experiences of Students from Abroad

All university students experience stress, but overseas students have additional strain because of higher financial obligations, less social support, language hurdles, and cultural acclimatization [15]. Relocating abroad is a risk to one’s health since students who are away from home frequently experience psychological disorders, including homesickness and loneliness [17]. International students experience sadness, stress, and anxiety due to these problems, including acculturative stress [18,19,20,21,22,23]. 

#### 1.2.1. International Students and Language Barriers

Language barriers and cultural behavioral variations further impact students’ relationship experiences abroad [24,25,26]. Language can be a communication challenge for students from different countries, resulting in exclusionary behaviors restricting social and academic relationships [21,27]. As a result, international students frequently make friends with classmates with comparable languages and cultural backgrounds, which helps them feel at ease and connected [17]. Although these networks of international friends are essential, they could prevent local students from developing deep relationships [28].

#### 1.2.2. International Students and Loneliness

According to earlier studies, international students face a variety of difficulties, such as strains related to cultural adjustment and adaptation [29,30]. A prevalent adverse experience these individuals share is the sensation of isolation [31]. According to Jiang et al., the standard method of assessing loneliness involves asking people to answer questions about their subjective perceptions of not having fulfilling relationships with other people [32]. The results of the score represent different degrees of loneliness. Profound loneliness, defined as persistently high levels of loneliness, has been connected to several unfavorable health outcomes, including eating disorders, anxiety, stress, depression, and alcohol abuse [33]. International students who are studying abroad often experience feelings of loneliness because they feel cut off from their home countries and the country they are studying in [34,35]. 

#### 1.2.3. International Students and Homesickness

**Figure 1:**
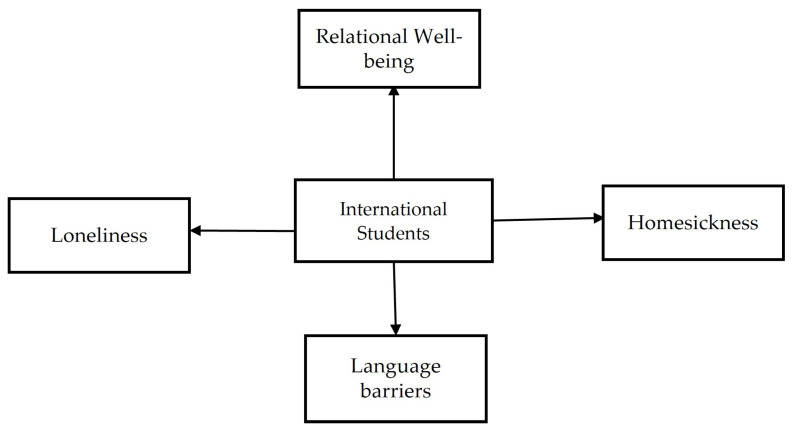
Conceptual framework for study.

According to Thurber and Walton, homesickness is the discomfort or impairment brought on by an actual or impending separation from one’s home [36]. Persistent thoughts of home and attachment objects are part of the cognitive aspect of homesickness [37]. People who are homesick frequently exhibit withdrawn behavior, a mix of anxious and depressed symptoms, and trouble focusing on subjects unrelated to their home [38]. When homesickness is moderate, it can promote healthy attachment behaviors like getting in touch with loved ones again and helping people to learn coping mechanisms [39]. It is important to remember that homesickness is a phenomenon that almost everyone experiences to some extent when they are away from home. Severe homesickness, however, can be crippling and upsetting. Thus, combining all of those factors as a central research theme, the researcher has combined and made a framework, as shown in Figure 1. 

### 1.3. Research Gap

There is a deficit in the particular investigation of elements impacting relational well-being at Hohai University, despite the development in research on relational well-being among international students and international studies. Although previous research provides a broad picture of the difficulties encountered by international students in various contexts, it does not explicitly analyze the unique dynamics present in the Hohai University community. By examining the effects of cultural uniqueness, the campus environment, interpersonal dynamics, and support networks on the relational well-being of international students at Hohai University, this study aims to close this gap. Hohai University is a research area that provides a culturally diverse setting, making it suitable for studying the relational well-being of international students. The findings can help other universities improve support services and foster better well-being and academic outcomes for their international student communities. In this unique cultural and educational setting, the study seeks to offer insightful information for improving general well-being and customizing support systems.

## 2. Methodology

### 2.1. Case Study Approach

The relational well-being of international students at Hohai University was thoroughly examined using the mixed-methods approach (quantitative/qualitative) and descriptively using Hohai University as a case study approach. According to Brannen and Coram, this method is more appropriate for gaining justifiable consequences in social sciences research [40]. The design facilitates a comprehensive comprehension of the distinct circumstances, encounters, and obstacles international students encounter in their interpersonal dynamics.

### 2.2. Sampling and Data Collection

The study aimed to explore the experiences and well-being of international students at Hohai University using a mixed-methods approach. A sample of 150 international students was strategically selected to ensure diverse representation across various academic programs, countries, and specializations. A well-structured questionnaire was utilized for the quantitative component, employing random sampling to enhance generalizability. The questionnaire, designed to be valid and reliable, featured a 10-point Likert scale to capture a wide range of responses.

In addition, qualitative data were collected through in-depth, face-to-face interviews with ten international students. These semi-structured interviews allowed the researcher to better understand the students’ relationship experiences, challenges, and coping mechanisms. This qualitative approach provided rich, detailed insights that complemented the broader quantitative findings. By employing quantitative and qualitative methods, the study aimed to comprehensively understand the international students’ experiences at Hohai University. The quantitative data provided a broad overview of trends and patterns, while the qualitative data offered in-depth insights into individual experiences. This mixed-methods approach ensured a robust analysis, enhancing the reliability and validity of the study’s findings. 

### 2.3. Data Analysis

The descriptive analysis was performed using SPSS, including the regression analysis, percentage, minimum, maximum, mean, and standard deviation. Qualitative data were collected using in-depth interviews involving women/men participants. However, qualitative data were analyzed manually by following the prescribed steps (data recording, transcribing, translating, refining, coding, and de-coding), where NVIVO software was used to generate a word cloud to enhance the accuracy of the results. 

Validity and reliability contribute to reducing usability problems, since usability offers participants an easy administration of an instrument [41]. According to Goswami, reliability is defined as the consistency of a scale that can be assessed using Cronbach’s alpha, and validity may be guaranteed using Confirmatory Factor Analysis (CFA) utilizing statistical software [42]. Accordingly, Average Variance Extracts (AVEs) with a value of 0.40 or more are regarded as having good validity and should be considered for additional investigation. Using CFA, a researcher can determine if a set of observed variables have a link [43]. The constructions’ reliability is shown in Table 1, which displays good results, because the Cronbach’s alpha value should not be less than 0.70.

## 3. Results

### 3.1. Descriptive Statistics of Background of the Respondents

The respondents’ primary background characteristics—with a particular emphasis on age—are displayed in the descriptive statistics table. The reported ages in Table 2 range from a minimum of 24 years to a maximum of 36 years, with a sample size of 150 respondents. The respondents’ mean age, or average age, is 28 years, representing the age distribution’s central tendency. There appears to be a moderate degree of variability in the reported ages, as indicated by the standard deviation of 5.7, which quantifies the dispersion of data around the mean. The respondent’s age profile is briefly summarized in this statistical summary, showing the dataset’s central tendency and age distribution.

The respondents’ demographics are shown in the Table 3; the data were retrieved from the both genders with a male to female student ratio of 70.7 to 29.3. Regarding academic endeavors, 47.3% of participants are enrolled in Master’s programs with a frequency of 71, 44.7% of participants are in PhD programs with a frequency of 67, and 8.0% of participants are Bachelor’s students. Regarding financial aid, 17.4% of participants receive scholarships from the China Scholarship Council (CSC), and the remaining 75.3% receive university scholarships. Some self-funded students were also observed at 7.3%. The percentages reveal the distribution of academic levels and scholarly areas among the sample of 150 respondents. 

The results of a regression analysis examining how international students perceive various factors influencing their well-being using a Likert scale are presented in Table 4. The unstandardized coefficients indicate the dependent variable’s change for every unit shift in the predictor variable. Relational well-being, in particular, shows a positive correlation (0.146) with a T value of 3.6807, highlighting its importance in influencing foreign students’ perceptions. With a robust positive effect of 0.143 and a high T value of 5.931, homesickness clearly has a significant impact. The language barrier also contributes significantly (0.125) with a T value of 4.378. On the other hand, loneliness has a negligible effect (0.011) with a T value of 0.847; its *p*-value of 0.368 suggests that it is not significant. The intercept term, 5.029, is the dependent variable’s expected value when none of the predictor variables is zero. To summarize, the standardized coefficients provide insight into the relative significance of each predictor, indicating that language barriers and homesickness are significant factors in influencing how international students are perceived.

Regarding the perception of the international students, the researcher utilized a Likert scale questionnaire. The Likert scale has been beneficial in social sciences research because it helps to gain perspective. A score of one means strongly disagree and ten means strongly agree. The survey’s findings, shown in Table 5, offer insightful information on the interpersonal experiences of international students at Hohai University. Most participants said they had no trouble connecting with nearby pupils, resulting in a solid mean score of M = 8.35; SD = 1.43. The average score for the institution’s assistance of international students’ social integration was M = 7.59; SD = 2.13, indicating a good response. However, the average score for the depth of friendships formed with peers from both domestic and international backgrounds was M = 5.73; SD = 1.89, which is slightly lower. Participants said Hohai University provided positive social experiences through various activities and events (M = 7.97; SD = 1.15) and fostered a welcome environment for intercultural discourse (M = 8.22; SD = 1.57). In connection to this, (M = 7.65; SD = 1.67), cultural unfamiliarity appeared as a considerable barrier. Additionally, a significant percentage of individuals reported experiencing discrimination because of their cultural background (M = 7.32; SD = 2.35). On the other hand, the average score for views of acceptance and understanding from the university community was lower, at M = 5.13; SD = 1.45. At an average of M = 5.89; SD = 2.15, the interaction and communication among the foreign student community was deemed rather suitable. Most respondents (M = 6.84; SD = 2.57) felt at ease asking university staff for help or guidance with social and relationship-related concerns. Additionally, the average score for the capacity to communicate with local people was comparatively lower (M = 3.57; SD = 2.23), showing the language barrier to a greater extent, although a smaller percentage (M = 3.16; SD = 1.15) regretted being accepted to Hohai University. These results highlight the university’s assets in fostering relationships and cross-cultural communication while offering suggestions for deepening friendships and solving issues among a portion of the student body.

### 3.2. Relational Well-Being from International Student’s Context 

To gain in-depth knowledge, the researcher analyzed the responses of participants through qualitative parameters, keeping in mind the objectives. The NVIVO software was used to analyze the results using word cloud, where the more significant words show the repetition of the phrase. A number of important elements foster international students’ positive relationship well-being. An essential factor in social integration is the development of friendships and involvement in cross-cultural activities, which provide participants with a sense of community and support [44]. Accessible counseling services and mentorship programs offer crucial support, helping students overcome psychological and academic obstacles. Peer networks, familial ties, and language assistance all contribute to improving the social experience. Positive and inclusive environments are also fostered by organizations that offer training to promote cultural competency and develop inclusive policies and housing arrangements [45]. Through these services, academic institutions help overseas students form solid social networks and ensure their success throughout their educational careers.

### 3.3. Interactions with Other International Students at Hohai University in Terms of Relationship

**Figure 2:**
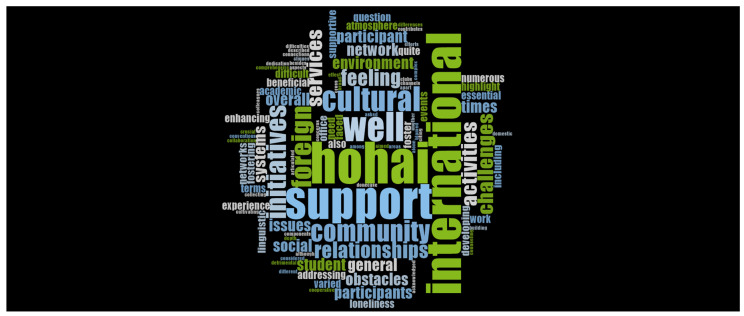
Relational Well-Being. Source: authors’ own analysis using NVIVO.

**Figure 3:**
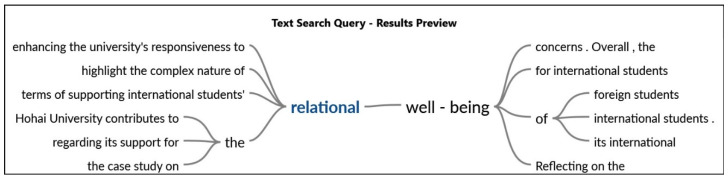
Correlation of the keywords with statements.

The Figure 2 shows the relationship of the words with statements provided by the participants. Regarding the inquires, one respondent stated that, *developing* ties with other international students at Hohai University has been a varied experience for me, filled with both good times and sometimes challenging times. Interacting with colleagues from different nations through planned university functions and cooperative research initiatives has enabled a broad cultural interchange and established a helpful network. Additionally, another participant highlighted that although there are times when overcoming linguistic and cultural obstacles might be difficult, Hohai University’s general environment is friendly and supportive of developing relationships. The institution’s dedication to cultivating an international community via activities and support services has been crucial in establishing a feeling of inclusion and enhancing the international student encounters as shown in Figure 3.

### 3.4. Support Systems or Networks at Hohai University

**Figure 4:**
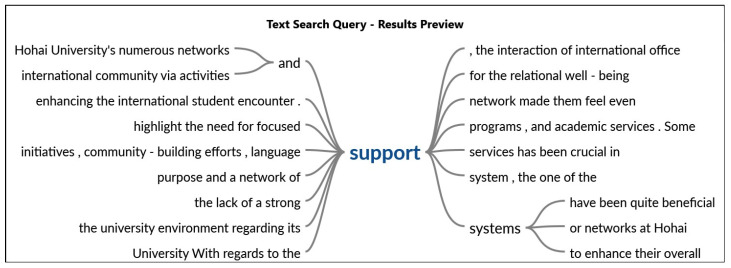
Support system of Hohai University for international students.

Regarding the support system as shown in Figure 4, one participant articulated that Hohai University’s numerous networks and support systems have been quite beneficial and have improved general well-being. For international students, the foreign Student Office facilitates a seamless transfer by offering vital services including orientation events and information on academic and everyday topics. The university’s social activities and cultural exchange initiatives foster a sense of community and help fight feelings of loneliness. Additionally, another participant said that participating in community events and activities, including sports, clubs, and volunteer work, helps foster a feeling of purpose and a network of support. The interaction of international office staff and the collaboration in activities are pretty beneficial. Apart from that, the university’s counseling services have been essential in addressing issues related to emotional and psychological health. These many resources work together to provide a welcoming and enriching atmosphere for foreign students at Hohai University.

### 3.5. University Environment in Terms of Supporting International Students’ Relational Well-Being

**Figure 5:**
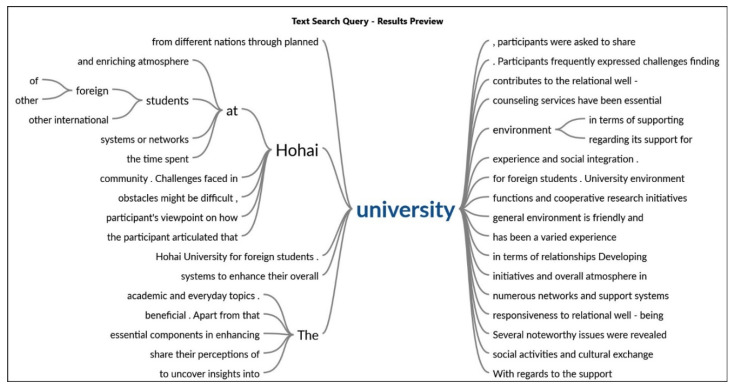
University and international students.

Reflecting on the time spent at Hohai University, participants were asked to share their perceptions of the university environment regarding its support for the relational well-being of international students, highlighted in Figure 5. The question aimed to uncover insights into the university’s initiatives and overall atmosphere in fostering or hindering relationships among students. Responses varied, highlighting supportive initiatives, community-building efforts, language support programs, and academic services. Some participants acknowledged areas for improvement, suggesting the need for more targeted initiatives addressing the unique challenges international students face. The feedback mechanisms and channels for collecting input were also essential in enhancing the university’s responsiveness to relational well-being concerns. Overall, the question provided a comprehensive understanding of the participant’s viewpoint on how Hohai University contributes to the relational well-being of its international student community.

### 3.6. Challenges Faced in Hohai University

**Figure 6:**
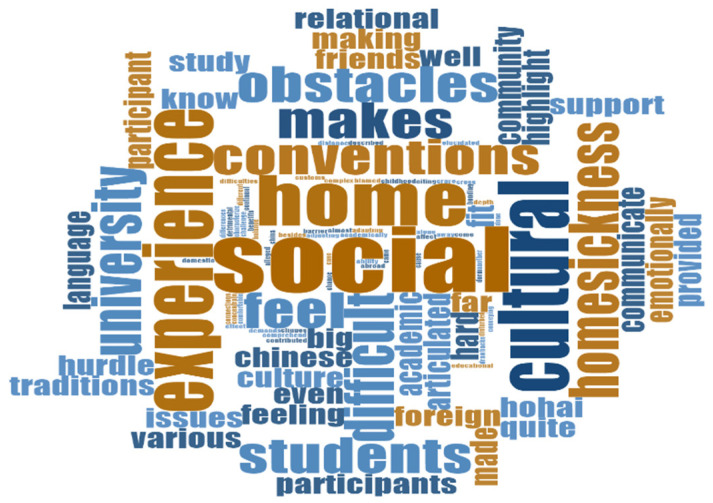
Word cloud of challenges/barriers in Hohai University. Source: authors’ own analysis using NVIVO.

Several noteworthy issues were revealed by the in-depth interviews performed as part of the case study on the relational well-being of international students at Hohai University. Participants frequently expressed challenges about finding and keeping meaningful connections, and they blamed linguistic obstacles, cultural differences, and a generalized feeling of loneliness for these issues as highlighted in Figure 6. One participant said that the language barrier has been the big hurdle to communicate with dorm staff because conveying thoughts is quite challenging. As elucidated, a person’s ability to integrate into the social and academic facets of their school experience in China might be hampered by their inability to comprehend and communicate in a language other than their mother tongue. This can cause feelings of isolation. 

**Figure 7:**
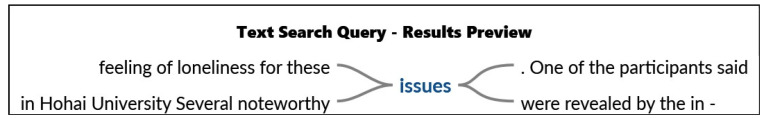
Issues regarding distance.

Additionally, homesickness has also emerged to be a hurdle in such an environment. One student stated the following: I am pretty disturbed being separate from home because it’s almost been one year being such distance that not only makes us feel our home but also the bonding that we can’t get here as shown in Figure 7. Another participant alleged that, throughout my study abroad experience, I had struggled emotionally to get over homesickness. Now and again, being away from my comfortable surroundings of home, family, and friends makes me feel lonely and homesick for the community I used to know. Adapting to various habits, traditions, and social conventions while navigating my educational path in a different nation has provided enrichment and emotional obstacles, contributing to periods of homesickness. As articulated by one respondent, being far from home has made me crave the things that are familiar and characterize my childhood, serving as a continual reminder of how far I’ve come both emotionally and physically from where I came from. Not only can homesickness affect my mental health, but it also sometimes makes it difficult for me to concentrate on my academic goals, making it difficult to strike a balance between the demands of both. 

**Figure 8:**
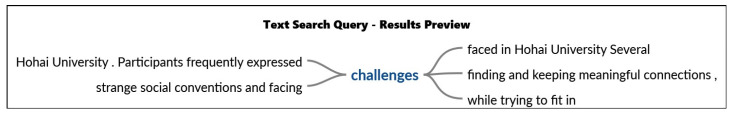
Challenges faced by the international students.

Moreover, the lack of a strong support network made them feel even more alone, which had a detrimental effect on their general well-being. Interviewees also emphasized difficulties making friends with domestic students, citing pre-existing cliques and cultural unfamiliarity as obstacles to fostering cross-cultural relationships. Numerous pupils described feeling overpowered by strange social conventions and facing challenges while trying to fit in with the neighborhood, demonstrated in Figure 8. As articulated by one participant, being in a foreign culture has proved to be a big challenge for me academically. There have been benefits and drawbacks to adjusting to various traditions, customs, and social conventions. I’ve occasionally felt lost since I don’t know enough about some parts of Chinese culture, which has motivated me to strive to understand and fit in with the community. Even though it has been difficult, this unusual experience has also allowed me to grow myself by encouraging flexibility and providing a more comprehensive view of the rich cultural landscape within my Chinese educational setting. These results highlight the complex nature of relational well-being for international students and the need for focused support systems to enhance their overall university experience and social integration.

## 4. Discussion

The study examines the interpersonal well-being of international students at Hohai University, analyzing issues such as homesickness, language obstacles, and loneliness, as well as their influence on students’ experiences and well-being. The study employs a mixed-methods methodology, integrating the quantitative data evaluated using SPSS and qualitative data examined utilizing NVIVO. This discussion consolidates the findings and places them in the context of the current literature, providing insights into the interpersonal dynamics and difficulties experienced by international students. The study demonstrates that homesickness substantially impacts the relational well-being of international students, as evidenced by a high T value of 5.931 and a strong positive effect of 0.143. This discovery is consistent with prior research emphasizing homesickness as a widespread problem among overseas students [36,37]. Homesickness can present itself as anxiety, despair, and impaired focus, leading to adverse impacts on students’ academic achievements and social assimilation [10]. The study highlights the necessity of implementing specific interventions to assist students in managing homesickness, such as counseling services and peer support groups, which have demonstrated their effectiveness in various situations [46]. Language limitations were seen as a significant obstacle, with a notable positive effect (0.125) and a T value of 4.378. Impediments to communication can impede academic achievement and social connections, resulting in a sense of exclusion [24]. This study affirms previous research by illustrating that language proficiency plays a pivotal role in the effective assimilation of international students [28]. Institutions can alleviate these obstacles by implementing language support programs and providing intercultural training for students and staff [45]. Surprisingly, loneliness had a minimal effect on relationship well-being (T value: 0.847), indicating that it is not a significant predictor in this situation. This finding contradicts prior research that repeatedly highlights loneliness as a significant concern for international students [12,14]. An explanation could be the efficacy of social integration programs at Hohai University, which may alleviate feelings of loneliness. Nevertheless, qualitative findings emphasize loneliness as a subjective experience, underscoring the significance of activities that foster community-building and support situations [15]. The study revealed that students usually consider Hohai University as offering a conducive environment for intercultural dialogue and social integration, as evidenced by the high average ratings for institutional help (M = 7.59) and intercultural atmosphere (M = 8.22). Perceptions play a vital role in maintaining healthy relationships, since the presence of supporting institutional practices can improve students’ feelings of belonging and overall satisfaction [45,46]. Nevertheless, some areas can be improved, namely cultivating more profound friendships and improving communication with local pupils, as indicated by lower average scores that highlight current difficulties. Qualitative data highlight the significance of solid support structures, such as the Foreign Student Office and counseling services, in enhancing relational well-being. These findings align with the existing literature that highlights the significance of institutional support in aiding with the adjustment of international students [18]. The participants also emphasized the presence of cultural obstacles and the necessity for more inclusive approaches to overcome the differences between domestic and international students. This aligns with the demand for comprehensive solutions to improve cross-cultural interactions.

## 5. Conclusions

This study emphasizes how important it is to foster good relationship well-being to enhance international students’ overall Hohai University experience. On behalf of the responses, the researcher concluded that the atmosphere and the interaction of the academic office were entirely satisfactory in terms of events, sports, as well as related matters. Additionally, the researcher also concluded that feelings of loneliness, homesickness, culture adoption, and language barriers seemed to be the challenges in the study area. The knowledge acquired can help create focused programs that address the unique difficulties encountered by international students, creating a more accommodating and encouraging campus community. This study adds to our understanding of relational well-being in the context of experiences for overseas students and may be helpful in other contexts. Hopefully, these results will stimulate continued efforts at Hohai University and other universities with varied student populations to improve the well-being of international students.

## Figures and Tables

**Table 1:** Validity and reliability of the data.

Appendix-I	Concept/Variable	No. of Items	Cronbach’s Alpha		CFA
			Pre-Test (n = 20)	Final Test (n = 150)	AVE ≥ 0.40
Reliability	Relational Well-Being	08	0.69	0.70	0.49
	Homesickness	06	0.71	0.72	0.72
	Language Barrier	07	0.68	0.71	0.53
	Loneliness	06	0.70	0.72	0.58

**Table 2:** Age of the respondents.

	N	Minimum	Maximum	Mean	Std. Deviation
Age	150	24.00	36.00	28.7500	5.78188

**Table 3:** General characteristics of the respondents.

	Frequency	Percentage
GENDER		
Male	106	70.7
Female	44	29.3
	**EDUCATION**	
Master	71	47.3
PhD	67	44.7
Bachelors	12	8.0
	**SCHOLARSHIP**	
CSC	26	17.4
University Scholarship	113	75.3
Self-Funded	11	7.3
	**Stay In Hohai**	
Six Months or Less	38	25.3
1 Year	76	50.7
2 Years	36	24.0

**Table 4:** Relational well-being and challenges of international students using regression analysis.

	Unstandardized Coefficients	Standardized Coefficients	SE	T Value	*p* Value	2.50%	97.50%
Relational Well-being	0.146	0.129	0.036	3.6807	0	0.069	0.219
Homesickness	0.143	0.201	0.026	5.931	0	0.094	0.179
Language Barrier	0.125	0.189	0.025	4.378	0	0.087	0.154
Loneliness	0.011	0.03	0.012	0.847	0.368	−0.016	0.040
Intercept	5.029	0	0.318	14.989	0	4.387	5.583

**Table 5:** Perception of international students in Hohai University.

S.No	Items	No	Minimum	Maximum	Mean	S.D
01	At Hohai University, I have no trouble making connections with local students.	150	2.00	10.00	8.3546	1.43507
02	The institution offers sufficient assistance to facilitate the social integration of overseas students.	150	1.00	10.00	7.5897	2.13409
03	I have made deep friendships with pupils from both domestic and foreign countries.	150	2.00	10.00	5.7325	1.89730
04	Hohai University provides a welcoming atmosphere for intercultural dialogue.	150	3.00	10.00	8.2156	1.15692
05	International students benefit from the social experiences provided by the university’s programs and events.	150	2.00	10.00	7.9673	1.57349
06	The degree of engagement and communication among the foreign student population meets my needs.	150	2.00	10.00	5.8943	2.15629
07	Cultural unfamiliarity is an obstacle for me.	150	2.00	10.00	7.6543	1.67895
08	At Hohai University, I have encountered prejudice because of my cultural background.	150	3.00	10.00	7.3246	2.35862
09	I think people at Hohai University accept and understand my cultural background.	150	2.00	10.00	5.1325	1.45686
10	I feel at ease asking university employees for advice or assistance with social and relationship problems.	150	1.00	10.00	6.8390	2.57826
11	I have no trouble communicating with domestic pupils.	150	2.00	10.00	3.5664	2.23549
12	I have some regrets about being selected for Hohai University.	150	1.00	10.00	3.1563	1.15789

## Data Availability

The raw data supporting the conclusions of this article will be made available by the authors on request.
